# Pinocembrin Decreases Atrial Fibrillation Susceptibility in a Rodent Model of Depression

**DOI:** 10.3389/fcvm.2022.766477

**Published:** 2022-05-20

**Authors:** Qian Ran, Xiaoli Chen, Cui Zhang, Weiguo Wan, Tianxin Ye, Yazhou Sun, Xin Zhao, Shaobo Shi, Bo Yang, Qingyan Zhao

**Affiliations:** ^1^Department of Cardiology, Renmin Hospital of Wuhan University, Wuhan, China; ^2^Cardiovascular Research Institute, Wuhan University, Wuhan, China; ^3^Hubei Key Laboratory of Cardiology, Wuhan, China

**Keywords:** pinocembrin, depression, atrial fibrillation, electrical remodeling, oxidative stress

## Abstract

**Background:**

Depression is often comorbid with cardiovascular diseases and contributes to the development and maintenance of atrial fibrillation (AF). Ample research demonstrated that pinocembrin had protective effects on the neuropsychiatric and cardiovascular systems *via* its pharmacological properties. However, whether pinocembrin protects from AF in depression models is not known. The present research investigated antiarrhythmic effects of pinocembrin and the underlying mechanisms in depressed rats.

**Methods:**

One hundred and ten male Sprague Dawley rats were randomly divided into six groups: the CTL group (the normal rats administered saline), the CTP group (the normal rats administered pinocembrin), the MDD group (the depressed rats administered saline), the MDP group (the depressed rats administered pinocembrin), the MDA group (the depressed rats administered apocynin), and the MPA group (the depressed rats administered both pinocembrin and apocynin). Chronic unpredictable mild stress (CUMS) was performed for 28 days to establish the depression model. Pinocembrin was administered *via* gavage from Day 8 to Day 28, and apocynin was administered *via* intraperitoneal injection from Day 1 to Day 28. The effects were evaluated using behavioral measurements, *in vitro* electrophysiological studies, whole-cell patch-clamp recordings, biochemical detection, Western blot, and histological studies.

**Results:**

Pinocembrin treatment significantly attenuated the abnormality of heart rate variability (HRV), the prolongation of action potential duration (APD), the shortening of the effective refractory period (ERP), the reduction of transient outward potassium current (I_to_), and the increase in L-type calcium current (I_Ca–L_), which increase susceptibility to AF in a rat model of depression. Compared to the depressed rats, pinocembrin also increased the content of Kv4.2, Kv4.3, and atrial gap junction channel Cx40 and decreased the expression level of Cav1.2, which ameliorated oxidative stress and inhibited the ROS/p-p38MAPK pro-apoptotic pathway and the ROS/TGF-β1 pro-fibrotic pathway.

**Conclusion:**

Pinocembrin is a therapeutic strategy with great promise for the treatment of AF in depressed patients by reducing oxidative stress.

## Introduction

Atrial fibrillation (AF) is the most common persistent arrhythmia in clinics. It is closely related to increased morbidity, primarily from stroke and heart failure, and increased mortality (1). AF also independently increases total mortality in patients with and without the cardiovascular disease (2). The risk of AF increases with age, hypertension, coronary heart disease, diabetes, systemic inflammation, and oxidative stress (3).

Depression is a mental disorder characterized by lethargy and mental slowness, and it may be accompanied by psychomotor retardation symptoms, such as a lack of enthusiasm for normal activities (4). Depression is a significant public health problem and has a special impact on health when accompanied by cardiovascular diseases (5). It is widespread in the elderly population. Recent data showed that the evaluation of depressive symptoms is an essential part of effective treatment for patients with AF (6). Depression contributes to the development and maintenance of AF and creates an environment that is conducive to AF (7).

Pinocembrin (5,7-dihydroxyflavanone) is a flavonoid that exhibits extensive pharmacological activities, including anti-inflammatory, antioxidant, and antimicrobial activities (8). Pinocembrin exhibited neuroprotective effects against cerebral ischemia/reperfusion injury (9), improved cognition, and protected the neurovascular unit in Alzheimer’s disease (10). Pinocembrin mitigated chronic unpredictable mild stress (CUMS)-induced depressive symptoms by alleviating oxidative stress (11). Recent studies showed that pinocembrin also exhibited cardioprotective effects. Pinocembrin decreased the risk of ventricular arrhythmias and reduced the myocardial infarction area in the myocardial ischemia-reperfusion rats (12). Pinocembrin decreased AF susceptibility in the myocardial infarction rats (13) and reduced ventricular fibrillation (VF) susceptibility in the depressed rats (14). However, whether pinocembrin alleviates AF susceptibility in the depressed rats is not clear.

The present research hypothesized that pinocembrin would mediate antiarrhythmic effects by ameliorating oxidative stress responses in the CUMS-induced depressed rats, which is a recognized depression model (15).

## Materials and Methods

### Animals

One hundred and ten male Sprague Dawley rats (200 ± 20 g) were randomly divided into six groups: (i) the CTL group, in which saline was administered *via* gavage from Day 8 to Day 28 (*n* = 20); (ii) the CTP group, which received 10 mg/kg pinocembrin *via* gavage from Day 8 to Day 28 (*n* = 20); (iii) the MDD group, which was subjected to CUMS for 28 days and received saline *via* gavage from Day 8 to Day 28 (*n* = 20); (iv) the MDP group, which was subjected to CUMS for 28 days and received 10 mg/kg pinocembrin *via* gavage from Day 8 to Day 28 (*n* = 20); (v) the MDA group, which was subjected to CUMS and received intraperitoneal injections of apocynin (a potent antioxidant, 3 mg/kg) for 28 days (*n* = 15); and (vi) the MPA group, which was subjected to CUMS and received both pinocembrin and apocynin (*n* = 15). The dose and the duration of administration were based on previous work (11, 16).

### Depression Model Building

The rats were subjected to one of the following triggers daily at random for 28 days: a lack of food or water for 24 h; 24-h lights on; 5-min tail clipping; 24-h cage tilt at 45°; small space for 2 h; forced swimming at 4 or 40°C for 5 min; 24-h wet bedding; and 24-h no bedding.

### Behavioral Measurements

#### Sucrose Preference Test

Each cage was supplied with two bottles of water. For the first 24 h, both bottles were filled with 1% sucrose. For the second 24 h, one of the bottles was filled with pure water, and then the two bottles were removed for 24 h. The rats were placed in separate cages supplied with two identical bottles containing 1% sucrose or pure water for 1 h. The placement of the bottles was switched after half an hour to avoid place preference. The two bottles were removed after a total time of 1 h.


$$\mathrm{Sucrose}\mathrm{preference}\left(\%\right)=\frac{\mathrm{sucrose}\,\mathrm{consumption}\,\left(g\right)}{\begin{array}{c} \mathrm{sucrose}\,\mathrm{consumption}\,\left(g\right)+\\ \mathrm{water}\,\mathrm{consumption}\,\left(g\right) \end{array}}$$

#### Forced Swimming Test

The FST assessed learned helplessness, which is a marker of depressive behaviors in rodents (17). The rats were placed separately in a lucent barrel, containing water for a total of 6 min. For the first 2 min, the rats were acclimated to the water, and the immobility time was calculated over the last 4 min.

#### Open Field Test

The OFT was performed to analyze the movement of the rats as described previously (14). Briefly, the rats were placed separately in the middle of an open field (100 cm × 100 cm × 50 cm), and the behavior of the rats was recorded and analyzed by a video-tracking system (EthoVision 3.0, Noldus) for 5 min. The traveling distance, average velocity, and the number of rearing events (the rat standing with its two forepaws lifted or climbing the wall more than 1 cm above the floor) were recorded. A solution of 75% alcohol was used to clean the open field between tests to remove the influence of smell.

#### Body Weights

The rats were weighed weekly during the experiment.

### Heart Rate Variability Analysis

A 15-min surface electrocardiogram (ECG) was recorded and analyzed to obtain time-domain and frequency-domain parameters of HRV. The time-domain included the average of RR intervals (average RR), the standard deviation of RR intervals (SDRR), and the square root of the mean squared differences of successive RR intervals (RMSSD), and the frequency-domain included low frequency (LF) from 0.2 to 0.75 Hz and the high frequency (HF) from 0.75 to 2.50 Hz. The LF/HF ratio was calculated to evaluate the autonomic balance.

### Atrial Electrophysiological Parameters

#### Heart Isolation

The rats were anesthetized *via* an intraperitoneal injection of pentobarbital (60 mg/kg) and heparinized with sodium heparin (400 units). The hearts were captured and instantly cannulated into a Langerdorff perfusion system (AD Instruments). HEPES-buffered Tyrode solution was used to perfuse the isolated hearts according to a previous study (18).

#### Action Potential Duration

The atrial monophasic action potentials (MAPs) were recorded from the left atrial appendage (LAA) by two custom-made Ag-AgCl electrodes. To evaluate the atrial action potential duration (APD), an S1S1 stimulation protocol with 10 stimuli was performed at pacing cycle lengths (PCLs) of 250, 200, 150, and 100 ms. The APD was detected at 90% repolarization (APD_90_) and 50% repolarization (APD_50_).

#### Effective Refractory Period

A programmed S1S2 stimulation consisting of eight basic stimuli (S1) [cycle length (CL): 200 ms], followed by a preceding stimulus (S2), was used to obtain the atrial effective refractory period (ERP). The longest S2 interval that did not catch the atrium was defined as the ERP.

#### Atrial Fibrillation Susceptibility

Atrial fibrillation (AF) susceptibility was determined by six applications of 50-Hz burst pacing. AF was defined as AF occurrence and maintenance for at least 2 s.

The PowerLab system was used to record all of the above signals, and LabChart 8.0 software was used for analyses.

### Whole-Cell Patch Clamp Recordings

#### Isolation of Atrial Myocytes

The hearts were captured and cannulated as described previously (18). The hearts were perfused with Ca2+-free Tyrode solution. The atrial appendages were excised and cut into small pieces to obtain isolated atrial myocytes (19, 20). Atrial myocytes were adhered to the bottom of a groove and observed under an inverted microscope. A rupture patch clamp was used for ion currents recordings.

Ion currents were recorded from 6 cells in each group.

#### I_Ca–L_ Recording

I_Ca–L_ was stimulated using the following procedure: hold potential (HP) = −40 mV, 200-ms pulses of voltages between −50 and +60 mV in 10-mV steps prior to a 50-ms prepulse of −40 mV. Steady-state inactivation of I_Ca–L_ was stimulated by the double-pulse procedure: a 300-ms prepulse from −50 to +20 mV with 10-mV increments, followed by a fixed 300-ms test pulse of 20 mV. The recovery of I_Ca–L_ was measured using the double-pulse procedure, including two identical pulses (holding potential between −50 and +20 mV for 300 ms) from 30 to 480 ms in an increment of 30 ms (20).

#### I_to_ Recording

For I_to_ recording, CdCl2 was added to inhibit I_Ca–L_. I_to_ was activated using the procedure of 500-ms step depolarizations from an HP of −80 to +20 mV. The voltage-dependent inactivation of I_to_ was measured using the double-pulse procedure: a 1,000-ms prepulse from −100 to +30 mV with 10-mV increments followed by a fixed 50-ms test pulse of 40 mV. The recovery of I_to_ was examined using the double-pulse procedure: a 500-ms conditioning pulse of +40 mV was isolated from a 50-ms test pulse of −40 mV by a gradually prolonged recovery interval from 50 to 800 ms in 50-ms increments (20).

The activation conductance variables (I/I_max_) and the inactivation conductance variables (I/I_max_) were fitted to the Boltzmann distribution to obtain the half activation or inactivation voltage (V_1/2_). The time courses of recovery were measured by plotting the normalized peak test current as a function of the recovery intervals, which were further fitted to a monoexponential function.

### Biochemical Detection

Venous blood was centrifuged at 3,000 *g* at 4°C for 15 min. Serum was used to detect the concentrations of MDA, H2O2, GSH, and GSSH and the activity of SOD.

### Western Blot Analysis

Tissues from the left atrium (LA) were used for Western blotting, which was performed based on our previous study (20). Membranes were blotted with antibodies against NOX2 (1:1,000; Abcam), NOX4 (1:1,000; Abcam), CaMKIIδ (1:1,000, Abcam), p-CaMKIIδ (1:1,000, Bioss), p38MAPK (1:1,000, Bioss), p-p38MAPK (1:1,000, Bioss), Cav1.2 (1:1,000, Abcam), Kv4.2 (1:1,000, Abcam), Kv4.3 (1:1,000, Abcam), TGF-β1 (1:1,000, Abcam), and collagen I (1:1,000, Bioss). GAPDH (1:1,000, Servicebio) was used as a reference protein.

### Histological Analysis

Tissues from the LA were embedded in paraffin and cut into 5-μm sections. Sirius red staining was used to assess atrial fibrosis, and immunohistochemical staining was performed to measure the level of the gap junction channel Cx40. TUNEL staining was used to examine atrial cell apoptosis. Apoptotic index (AI) = the number of apoptotic cells/the number of nucleated cells.

### ROS Measurement

Frozen LA sections were stained with 5-mM DHE at 37°C for 30 min and observed by fluorescence microscopy to evaluate the fluorescence intensity of ROS.

Tissues used for patch clamp, Western blotting, histological analyses, and ROS measurements were obtained from non-perfused hearts.

### Statistical Analysis

Continuous variables are presented as the means ± SE, and ratios are shown as percentages. Student’s *T*-test or Pearson’s chi-squared test was used for comparisons between two groups, one-way ANOVA followed by Tukey’s multiple-comparisons test (variances were equal) and Welch’s one-way ANOVA, followed by Games-Howell’s multiple comparisons test (variances were unequal), which were used for comparisons between multiple groups, and a two-way ANOVA followed by Sidak’s multiple comparisons test was used for comparisons in body weight at different times and the APD at different PCLs between multiple groups. *p* < 0.05 was considered statistically significant.

## Results

### Pinocembrin-Alleviated Behavioral Measurements

**FIGURE 1:**
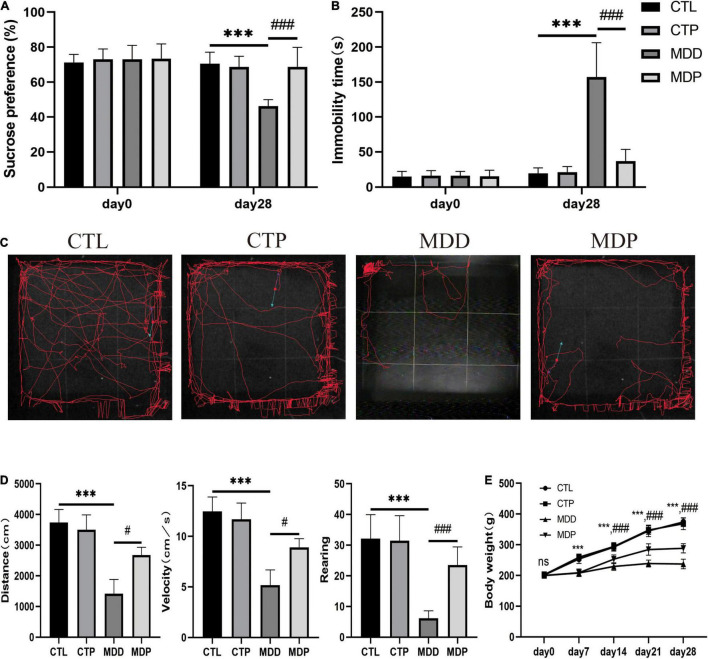
Behavioral measurements. **(A)** Sucrose preference before and after CUMS. **(B)** Immobility time before and after CUMS. **(C)** Typical images of the trajectory after CUMS. **(D)** Traveling distance, velocity, and number of rearing events. **(E)** Body weight during the experiment. *n* = 20 per group. ***p < 0.001 vs. CTL; #*p* < 0.05, ###*p* < 0.001 vs. MDD.

Before CUMS, there were no differences in sucrose preference, immobility time, or body weight between groups (Figures 1A,B,E). After 4 weeks of CUMS, the MDD group exhibited a remarkably reduced sucrose preference compared to the CTL group (46.17 ± 3.90 vs. 70.51 ± 6.58, *p* < 0.001). The FST revealed an increase in immobility time in the MDD rats compared to the CTL rats (157.10 ± 49.17 vs. 19.50 ± 7.88, *p* < 0.001). Figure 1C shows typical pictures of the trajectory of the groups on the OFT. Compared to the CTL rats, the MDD rats exhibited markedly reduced activities, including traveling distance, average velocity, and the number of rearing events (1,419 ± 462.1 vs. 3,741 ± 425.2, *p* < 0.001; 5.17 ± 1.52 vs. 12.47 ± 1.42, *p* < 0.001; 6.15 ± 2.46 vs. 32.10 ± 7.85, *p* < 0.001, respectively). All of these parameters were remarkably ameliorated in the MDP group treated with pinocembrin (Figures 1A–D). The body weights of the MDD rats were reduced during the 4 weeks compared with the CTL rats and were markedly increased after the administration of pinocembrin in the last 3 weeks (Figure 1E). The behavioral measurements did not differ markedly between the CTL rats and the CTP rats.

### Pinocembrin-Ameliorated Heart Rate Variability in Depressed Rats

**FIGURE 2:**
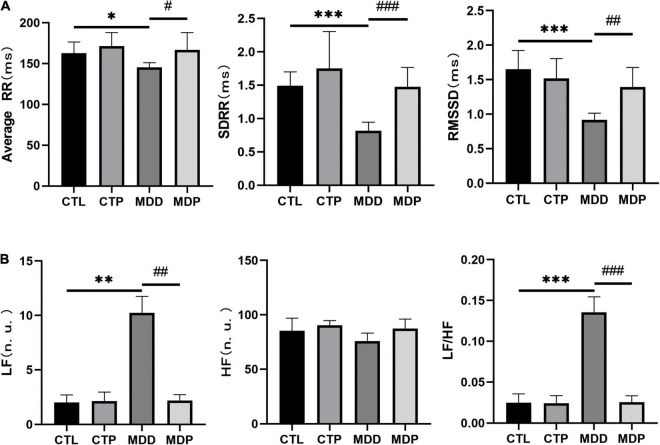
Statistical analyses of HRV. **(A)** Average RR, SDRR, and RMSSD. **(B)** LF, HF, and LF/HF. n = 8 per group. *p < 0.05, **p < 0.01, ***p < 0.001 vs. CTL; #*p* < 0.05, ##*p* < 0.01, ###*p* < 0.001 vs. MDD.

HRV includes time-domain and frequency-domain parameters. For the time domain, a reduction in the average RR, SDRR, and RMSSD was exhibited in the MDD rats compared to the CTL rats (145.3 ± 5.75 vs. 162.8 ± 13.71, *p* < 0.05;.82 ± 0.13 vs. 1.49 ± 0.21, *p* < 0.001;.92 ± 0.10 vs. 1.65 ± 0.27, *p* < 0.001, respectively, Figure 2A). For the frequency domain, LF and LF/HF were apparently increased in the MDD rats relative to the CTL rats (10.25 ± 1.50 vs. 2.00 ± 0.69, *p* < 0.01;.136 ± 0.019 vs. 0.025 ± 0.011, *p* < 0.001, respectively, Figure 2B). Significantly, all of the aforementioned parameters were ameliorated after pinocembrin administration (Figures 2A,B). There were no obvious differences in HRV between the CTL rats and the CTP rats, and HF did not differ significantly between the groups.

### Pinocembrin-Reversed Atrial Electrical Remodeling in Depressed Rats

**FIGURE 3:**
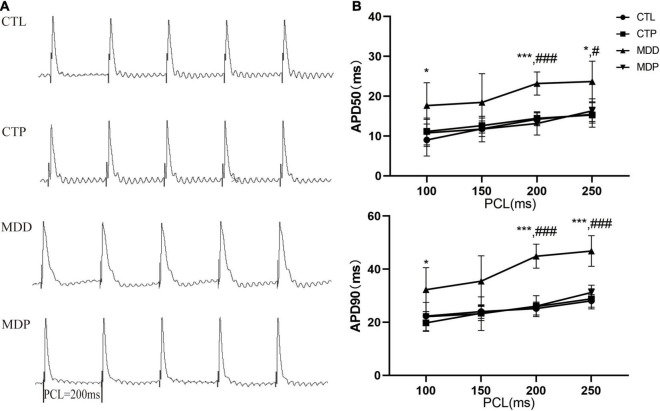
Action potential duration. **(A)** Typical recordings of APD at a PCL of 200 ms. **(B)** The spatial dispersions of APD_50_ and APD_90_. *n* = 8 per group. *p < 0.05, ***p < 0.001 vs. CTL; #*p* < 0.05, ###*p* < 0.001 vs. MDD.

Figure 3A shows the typical APDs obtained from the LAA in the four groups at a PCL of 200 ms. The APD_50_ and APD_90_ were obviously longer at PCLs of 100, 200, and 250 ms in the MDD rats than the CTL rats, but these changes were effectively reversed after pinocembrin treatment at PCLs of 200 ms and 250 ms (Figure 3B, all *ps* < 0.05).

**FIGURE 4:**
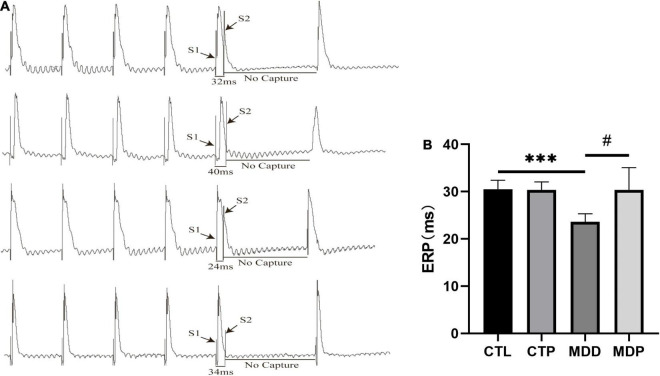
An effective refractory period. **(A)** Typical atrial ERPs. **(B)** Average ERP in the four groups. *n* = 8 per group. ***p < 0.001 vs. CTL; #*p* < 0.05 vs. MDD.

Figure 4A shows the typical atrial ERPs of the groups. The average ERP was markedly decreased in the MDD rats compared to the CTL rats (23.63 ± 1.69 vs. 30.50 ± 1.93, *p* < 0.001, Figure 4B) and was reversed by pinocembrin in the MDP group.

**FIGURE 5:**
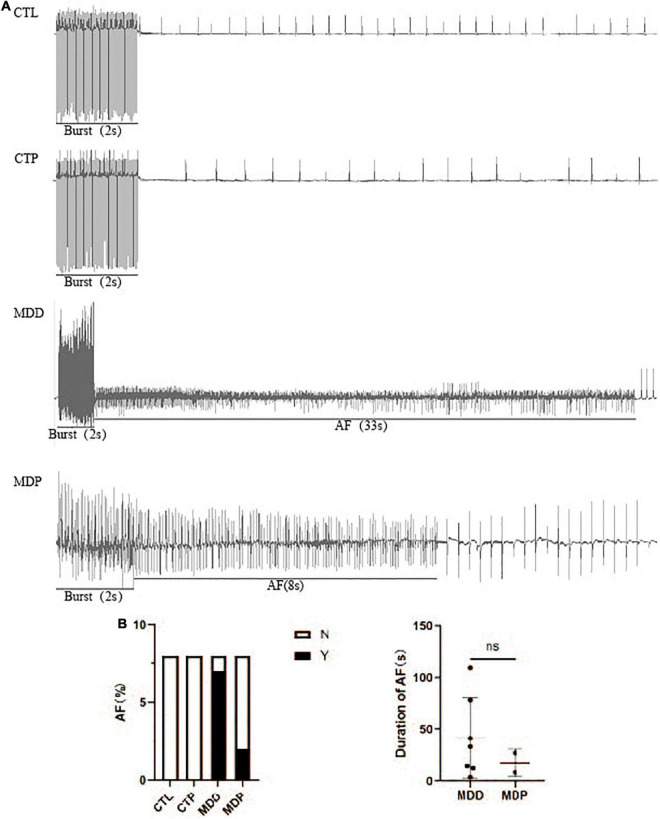
AF susceptibility. **(A)** Typical recordings of AF. **(B)** Inducibility and duration of AFs. *n* = 8 per group. ns, no significance.

Figure 5A shows the typical recordings of the burst pacing protocol. AF was induced in 7 of the 8 MDD rats and 2 of the 8 MDP rats but not in the 8 CTL rats or the 8 CTP rats (Figure 5B). However, the average duration of AF showed no apparent difference between the MDD rats and the MDP rats (41.49 ± 38.81 vs. 17.50 ± 13.44, *p* > 0.05, Figure 5B).

### Pinocembrin Increased I_to_ in Atrial Myocytes in Depressed Rats

**FIGURE 6:**
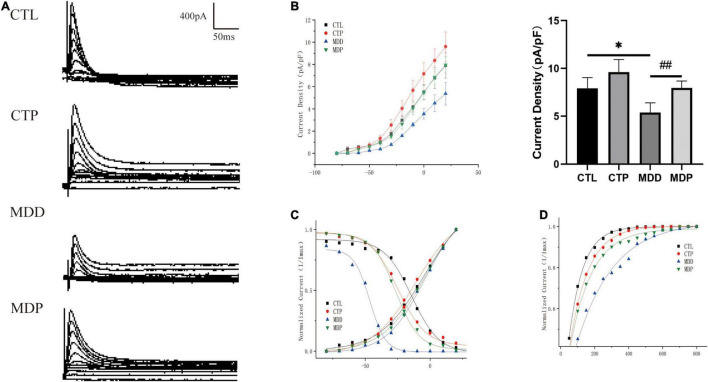
Electrophysiological characteristics of I_to_. **(A)** Typical I_to_ current traces. **(B)** The voltage-dependent current density (the I-V curve) and maximum current density of I_to_. **(C)** The voltage-dependent activation curve and the steady-state inactivation curve fitted to the Boltzmann distribution. **(D)** The recovery curve fitted to the monoexponential function. *n* = 6 per group. *p < 0.05 vs. CTL; ##*p* < 0.01 vs. MDD.

I_to_ is the outward K^+^ current that primarily contributes to the first phase of action potential repolarization. Figures 6A,B show typical I_to_ current traces and I-V curves. The maximum I_to_ current density was remarkably reduced in the MDD group versus the CTL group (5.37 ± 1.02 vs. 7.91 ± 1.14, *p* < 0.05) but was significantly reversed in the MDP group treated with pinocembrin (7.96 ± 0.72 vs. 5.37 ± 1.02, *p* < 0.01, Figure 6B). As shown in Figure 6C, the activation curve in the CTP rats was remarkably left shifted when compared to the CTL rats (−11.29 ± 1.13 vs. −4.78 ± 2.21, *p* < 0.001, Table 1), and it did not differ between the CTL rats, MDD rats, and MDP rats. The inactivation curve of the MDD rats was markedly left shifted compared to the CTL rats (−48.76 ± 1.31 vs. −14.22 ± 1.37, *p* < 0.001, Table 1) and was restored in the MDP rats. Reduced recovery after inactivation was observed in the MDD rats compared to the CTL rats (216.54 ± 14.61 vs. 83.02 ± 1.81, *p* < 0.001, Figure 6D and Table 1), and recovery was accelerated after pinocembrin administration.

**TABLE 1:** Activation, inactivation, and recovery properties of I_to_.

	CTL	CTP	MDD	MDP
**Activation**				
V1/2	−4.78 ± 2.21	−11.29 ± 1.13***	−4.62 ± 1.32	−3.70 ± 1.10
**Inactivation**				
V1/2	−14.22 ± 1.37	−24.82 ± 1.09	−48.76 ± 1.31***	−26.70 ± 0.64###
**Recovery**				
Time constant τ	83.02 ± 1.81	108.83 ± 1.80	216.54 ± 14.61***	129.69 ± 5.06###

*n = 6 per group.*

***p < 0.001 vs. CTL; ###p < 0.001 vs. MDD.

### Pinocembrin Inhibited I_Ca–L_ in Atrial Myocytes in Depressed Rats

**FIGURE 7:**
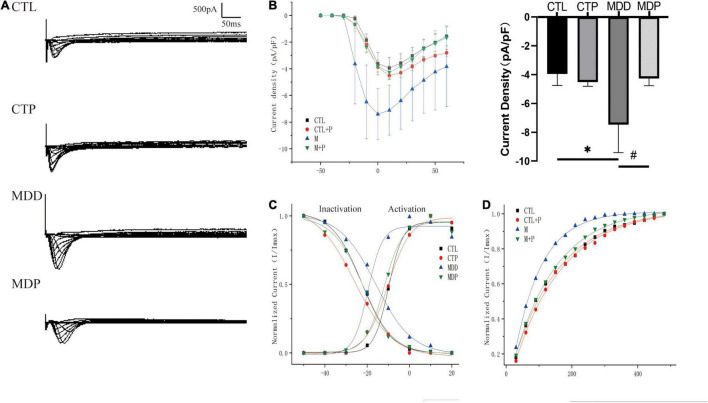
Electrophysiological characteristics of I_Ca–L_. **(A)** Typical I_Ca–L_ current traces. **(B)** The voltage-dependent current density (the I-V curve) and maximum current density of I_Ca–L_. **(C)** The voltage-dependent activation curve and the steady-state inactivation curve fitted to the Boltzmann distribution. **(D)** The recovery curve fitted to the monoexponential function. *n* = 6 per group. *p < 0.05 vs. CTL; #*p* < 0.05 vs. MDD.

Figures 7A,B show the typical I_Ca–L_ current traces and the I-V curves. The maximum current density was clearly increased in the MDD rats compared to the CTL rats (−7.47 ± 1.95 vs. −3.95 ± 0.81, *p* < 0.05, Figure 7B) but was markedly decreased after pinocembrin administration. As shown in Figure 7C, the activation curve was significantly left shifted in the MDD rats versus the CTL rats (−19.60 ± 0.96 vs. −9.88 ± 0.53, *p* < 0.001, Table 2) and was recovered by pinocembrin. The inactivation curve in the MDD rats was remarkably right shifted compared to the CTL rats (−15.89 ± 1.31 vs. −22.55 ± 0.38, *p* < 0.001, Figure 7C and Table 2) and was recovered in the MDP rats. The recovery time constant of I_Ca–L_ after inactivation is shown in Table 2. The recovery after inactivation was remarkably faster in the MDD rats (83.23 ± 1.84 vs. 144.81 ± 7.00, *p* < 0.001), and pinocembrin significantly retarded the recovery (131.87 ± 3.62 vs. 83.23 ± 1.84, *p* < 0.001, Figure 7D).

**TABLE 2:** The parameters of the activation, inactivation, and recovery kinetics of I_Ca–L_.

	CTL	CTP	MDD	MDP
**Activation**				
V1/2	−9.88 ± 0.53	−10.15 ± 0.67	−19.60 ± 0.96***	−12.04 ± 0.94###
**Inactivation**				
V1/2	−22.55 ± 0.38	−25.82 ± 0.93	−15.89 ± 1.31***	−22.08 ± 0.93###
**Recovery**				
Time constant τ	144.81 ± 7.00	149.11 ± 5.37	83.23 ± 1.84***	131.87 ± 3.62###

*n = 6 per group.*

***p < 0.001 vs. CTL; ###p < 0.001 vs. MDD.

### Pinocembrin Protected Against Oxidative Stress in Depressed Rats

**FIGURE 8:**
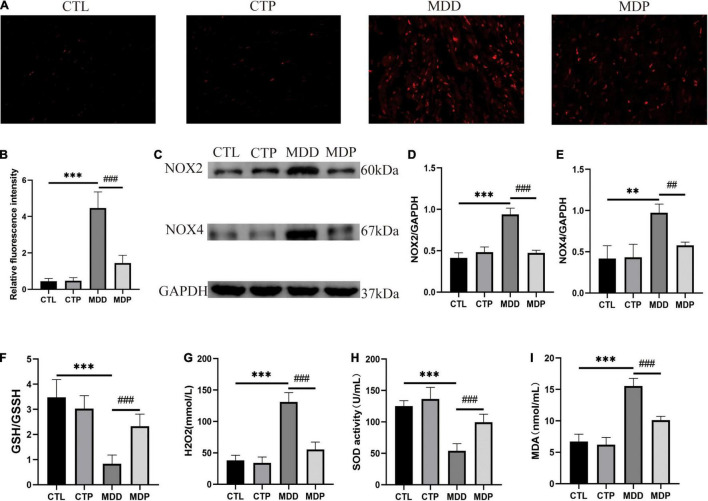
Oxidative stress. **(A)** Typical images of ROS. **(B)** Relative fluorescence intensity of ROS, *n* = 6 per group. **(C)** Immunoblotting of NOX2 and NOX4. **(D,E)** Quantitative analysis of NOX2 and NOX4, *n* = 5 per group. **(F–I)** GSH/GSSG, the concentration of H2O2, the activity of SOD, and the concentration of MDA in serum, *n* = 7 per group. **p < 0.01, ***p < 0.001 vs. CTL; ##*p* < 0.01, ###*p* < 0.001 vs. MDD.

Figure 8A shows typical images of ROS in the four groups. The level of ROS, the expression of NOX2 and NOX4 in the LA, and the concentration of H2O2 and MDA in serum were remarkably increased in the MDD rats versus the CTL rats (*p* < 0.05, Figures 8B–E,G,I), and the activity of SOD and the ratio of GSH/GSSG in serum were clearly reduced in the MDD rats (*p* < 0.05, Figures 8F,H). However, all of these indicators were reversed after pinocembrin treatment (*p* < 0.05). The oxidative stress responses showed no marked difference between the CTL and CTP rats.

### Pinocembrin Restored the Lack of Ion Channel Proteins and Cx40 in Depressed Rats

**FIGURE 9:**
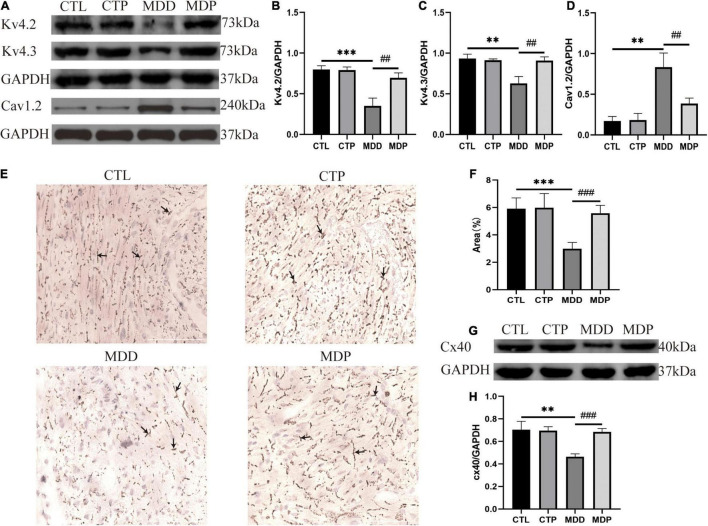
The content of ion channels and Cx40. **(A)** Immunoblotting of Kv4.2, Kv4.3, and Cav1.2. **(B–D)** Quantitative analysis of Kv4.2, Kv4.3, and Cav1.2, *n* = 5 per group. **(E)** Typical images of Cx40 immunohistochemical staining. **(F)** Percentage of the cx40 area, *n* = 6 per group. **(G)** Immunoblotting of Cx40. **(H)** Quantitative analysis of Cx40, *n* = 5 per group. **p < 0.01, ***p < 0.001 vs. CTL; ##*p* < 0.01, ###*p* < 0.001 vs. MDD.

The contents of Kv4.2 and Kv4.3 were remarkably reduced in the MDD rats, and Cav1.2 was highly expressed. However, the abnormalities in ion channel proteins were recovered by pinocembrin administration (Figures 9B–D). The main connexins of the heart include Cx40, Cx43, and Cx45, and Cx40 is primarily expressed in the atrium. Figure 9E shows representative images of Cx40 immunohistochemical staining in the four groups. The area of Cx40 was decreased in the MDD rats compared to the CTL rats (2.98 ± 0.47 vs. 5.91 ± 0.79, *p* < 0.001, Figure 9F) but was remarkably recovered after pinocembrin treatment. Western blotting further demonstrated the differences in Cx40 expression between the four groups (Figures 9G,H).

### Pinocembrin Suppressed the Content of p-p38 and p-CaMKIIδ and Ameliorated Atrial Cells Apoptosis in Depressed Rats

**FIGURE 10:**
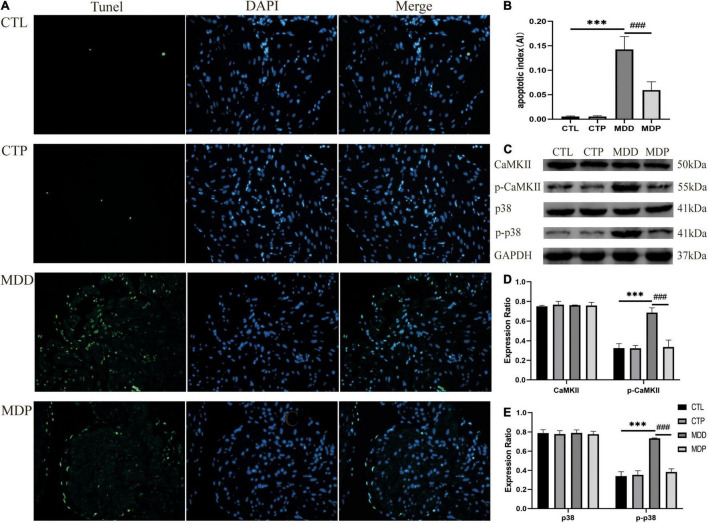
Atrial cell apoptosis and the levels of related proteins. **(A)** Typical images of TUNEL staining. Apoptotic cells exhibit green fluorescence, and nuclei show blue fluorescence. **(B)** The apoptotic index of the four groups, *n* = 6 per group. **(C)** Immunoblotting of CaMKII, p-CaMKII, p38, and p-p38. **(D,E)** Quantitative analyses of CaMKII, p-CaMKII, p38, and p-p38, *n* = 5 per group. ***p < 0.001 vs. CTL; ###*p* < 0.001 vs. MDD.

Figure 10A shows typical images of TUNEL staining in the four groups. Apoptotic cells were relatively increased in the MDD rats, and pinocembrin reversed the increase in the apoptotic cells. The MDD rats showed a higher apoptotic index (AI) than the CTL rats (0.142 ± 0.026 vs. 0.005 ± 0.001, *p* < 0.001, Figure 10B), and the AI was significantly decreased after pinocembrin administration. A remarkably increased level of p-p38 MAPK and p-CaMKIIδ was exhibited in the MDD rats, and the expression was reduced after pinocembrin treatment (Figures 10C–E). The contents of p38MAPK and CaMKIIδ did not differ significantly between the four groups.

### Pinocembrin Reduced the Content of TGF-β1, COL1 (CollagenI), and Ameliorated Atrial Fibrosis

**FIGURE 11:**
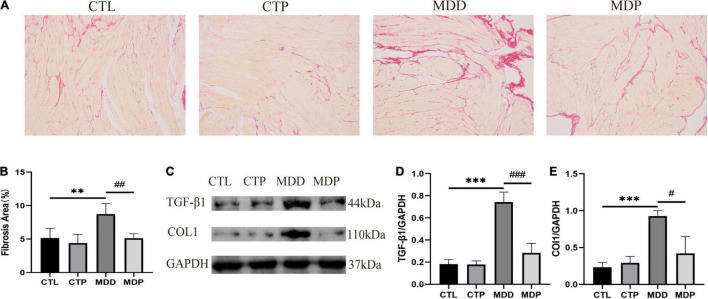
Atrial fibrosis and the expression of related proteins. **(A)** Typical images of Sirius red staining. **(B)** Percentage of the fibrosis area of the four groups, *n* = 6 per group. **(C)** Immunoblotting of TGF-β1 and COL1. **(D,E)** Quantitative analyses of TGF-β1 and COL1, *n* = 5 per group. **p < 0.01, ***p < 0.001 vs. CTL; #*p* < 0.05, ##*p* < 0.01, ###*p* < 0.001 vs. MDD.

Figure 11A shows typical images of Sirius red staining in the four groups. Myocardial interstitial collagen deposition and the contents of TGF-β1 and COL1 were remarkably increased in the MDD rats versus the CTL rats and were clearly reduced after pinocembrin administration (Figures 11B–E). No obvious difference was found between the CTL and CTP rats.

### Pinocembrin Reduced AF Susceptibility Through the Antioxidant Effect in Depressed Rats

**FIGURE 12:**
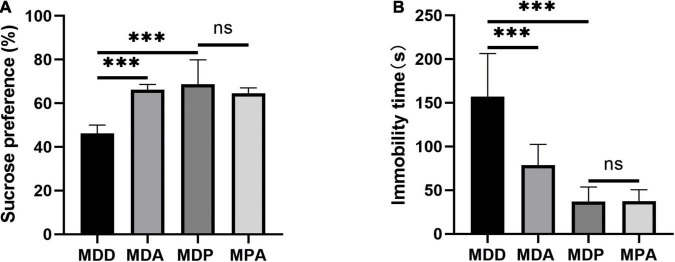
Behavioral measurements. **(A)** Sucrose preference after CUMS. **(B)** Immobility time after CUMS. ***p < 0.001 vs. MDD; ns, no significance.

**FIGURE 13:**
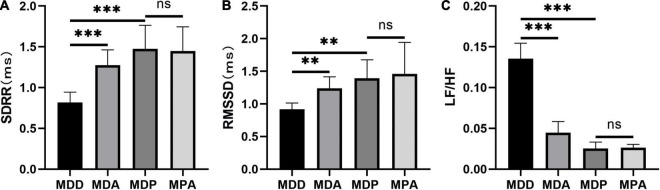
Statistical analyses of HRV. **(A)** SDRR. **(B)** RMSSD. **(C)** LF/HF. *n* = 8 per group. **p < 0.01, ***p < 0.001 vs. MDD; ns, no significance.

**FIGURE 14:**
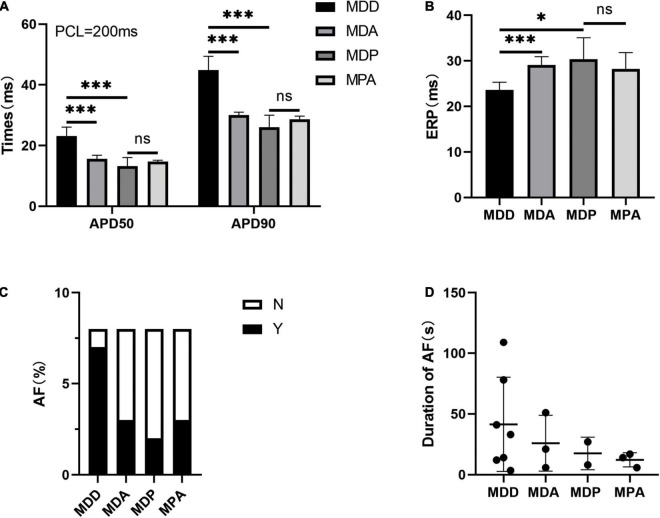
Atrial electrophysiological parameters. **(A)** APD_50_ and APD_90_ at a PCL of 200 ms. **(B)** Average ERP. **(C,D)** Inducibility and duration of AFs. *n* = 8 per group. *p < 0.05, ***p < 0.001 vs. MDD; ns, no significance.

**FIGURE 15:**
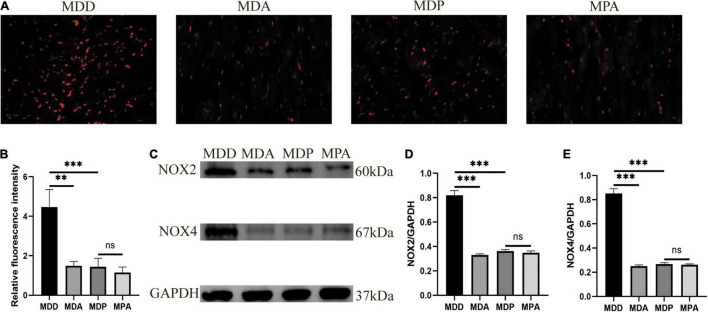
Oxidative stress. **(A)** Typical images of ROS. **(B)** Relative fluorescence intensity of ROS, *n* = 6 per group. **(C)** Immunoblotting of NOX2 and NOX4. **(D,E)** Quantitative analyses of NOX2 and NOX4, *n* = 5 per group. **p < 0.01, ***p < 0.001 vs. MDD; ns, no significance.

To further verify that the antioxidant effect of pinocembrin-ameliorated atrial arrhythmia and autonomic imbalance in depressed rats, the MDA group and the MPA group were included. After 4 weeks of CUMS, sucrose preference in the SPT and immobility time in the FST were improved in the MDA group compared to the MDD group (Figures 12A,B). However, no obvious difference was exhibited in depression-like behaviors between the MDP group and the MPA group. Figures 13A–C show that SDRR and RMSSD were increased, and LF/HF was decreased in the MDA group compared to the MDD group. However, there was no obvious difference in HRV between the MDP group and the MPA group. As shown in Figure 14A, APD_50_ and APD_90_ at a PCL of 200 ms were shortened in the MDA group compared to the MDD group. The average ERP was prolonged in the MDA group compared to the MDD group (Figure 14B). For AF susceptibility, AF was induced in 3 of the 8 MDA rats, which was remarkably decreased compared to the MDD group (Figure 14C). In addition, the average duration of AF showed no apparent differences between the four groups (Figure 14D). However, there was no significant difference in all these atrial electrophysiological parameters between the MDP group and the MPA group, suggesting that apocynin did not further increase the antiarrhythmic effects of pinocembrin. Figures 15A–E show that the level of ROS and the expression of NOX2 and NOX4 were remarkably reduced in the MDA group compared to the MDD group. There was no obvious difference in oxidative stress between the MDP group and the MPA group. Therefore, pinocembrin could reduce AF susceptibility through the antioxidant effect on depressed rats.

## Discussion

The present research indicated that pinocembrin ameliorated AF susceptibility and reduced oxidative stress in a rodent model of depression. Our research presented the following findings: (1) the inducibility of AF was increased in depressed rats; (2) pinocembrin treatment ameliorated depressive behaviors; (3) pinocembrin improved HRV and suppressed atrial autonomic remodeling in depressed rats; (4) pinocembrin ameliorated atrial electrical remodeling in depressed rats; (5) pinocembrin restored the reduced I_to_ and the increased I_Ca–L_ due to the improved content of Kv4.2 and Kv4.3 and the reduced level of Cav1.2 in depressed rats; and (6) pinocembrin attenuated atrial cell apoptosis and atrial fibrosis and improved the expression of Cx40 in depressed rats.

### Pinocembrin and Atrial Electrical Remodeling

The activation and recovery of action potentials (APs) contribute to the orderly operation of the myocardium (21). The recovery sequence includes repolarization and excitability recovery, which are expressed as APD and ERP, respectively (22). Imbalances in these electrophysiological indicators, such as prolonged or reduced APD, decreased ERP, or a combination of these changes, contribute to atrial arrhythmias (23). Consistent with previous research (18), our study showed that depressed rats exhibited prolonged APD and reduced ERP in the LA, which were restored by pinocembrin and resulted in reduced AF susceptibility.

Cav1.2 is responsible for the formation of I_Ca–L_ channels for inward Ca^2+^ currents, and Kv4.2 and Kv4.3 contribute to I_to_ channels for outward K^+^ currents. The balance of these two currents determines cardiac repolarization and refractoriness. I_to_ is the main current that underlies the early rapid repolarization of cardiac action potentials (24). I_to_ is rapidly activated and inactivated during cardiac depolarization and forms early rapid repolarization and the initial part of the plateau (25). Under pathological conditions, the physiological properties of I_to_ are changed, which leads to changes in myocardial repolarization and results in abnormalities in cardiac electrophysiological properties. The decreased I_to_ slows the early rapid repolarization, which prolongs APD and QT intervals and results in increased dispersion of repolarization and APD alterations (26). Prolonged APD and decreased I_to_ were demonstrated in the depressed rats (18, 19). I_Ca–L_ is the main current that underlies the plateau phase of cardiac action potentials. I_Ca–L_ determines the action potential duration, and increased I_Ca–L_ prolongs the plateau phase, which prolongs APD and QT intervals (27). Pathological myocardium remodeling is associated with sustained activation of I_Ca–L_ channels, which dominate calcium homeostasis (28). Abnormal Ca^2+^ handling may promote abnormal conduction and lead to reentry and other AF-related remodeling (29). Increasing intracellular Ca^2+^ activates calmodulin-dependent protein kinase II (CaMKII), and sustained activation of this enzyme results in autophosphorylation (30). p-CaMKII is closely associated with an arrhythmogenic phenotype. Our research showed an obvious reduction in the contents of Kv4.2 and Kv4.3 and increased Cav1.2 and p-CaMKII in the depressed rats. However, pinocembrin reversed the abnormalities of these indicators.

### Pinocembrin and Heart Rate Variability

SDRR and RMSSD represent the parasympathetic element of HRV (31). LF/HF is an indicator of autonomic balance. LF represents the sympathetic component, and HF represents the parasympathetic component (31). The parasympathetic system mediates the relationship between mental illness and cardiovascular events (32). Decreased parasympathetic activity increases the incidence of cardiovascular events and is associated with self-regulation disorder, poor social engagement, and lower mental resilience, which may result in the formation and progression of depression (33). Our study showed that parasympathetic activity was remarkably reduced in depressed rats and ameliorated by pinocembrin.

### Pinocembrin and Atrial Structural Remodeling

Atrial fibrosis disturbs cardiac electrical continuity, increases conduction heterogeneity, and reduces conduction speed, which ultimately leads to the pathogenesis and maintenance of AF. Interactions between fibroblasts and cardiomyocytes may contribute to ectopic activity and reentry. Therefore, atrial fibrosis is directly involved in the formation and maintenance of focal and reentry arrhythmias. Apoptosis is associated with the postnatal morphogenesis of the sinus node, atrioventricular node, and His bundle, and it is related to the pathogenesis and pathophysiology of cardiomyopathy, paroxysmal arrhythmia, and conduction disorder. Another potential mechanism of AF is the loss of Cx40, which is a major component of the atrial gap junction channel. Gap junction channels provide functional pathways and promote rapid diffusion of the action potential throughout the myocardium (34). Therefore, loss of Cx40 may prolong activation latency, lead to conduction block, and ultimately promote AF. The depressed rats in our study exhibited significantly increased atrial fibrosis and atrial cell apoptosis and decreased expression of Cx40. However, pinocembrin reversed all of these abnormalities.

### Pinocembrin and Oxidative Stress

Oxidative stress is characterized by an imbalance between the production and removal of reactive oxygen species (ROS) and impairs the ability to restore the damage to proteins, lipids, and DNA caused by reactive intermediates. Oxidative stress promotes the pathophysiology of cardiovascular diseases, including hypertension, cardiomyopathy, heart failure, and coronary artery disease, and psychiatric disorders, such as depression and Alzheimer’s disease. A previous study found that the levels of inflammatory and oxidative stress indicators in blood were significantly increased in subjects with depression compared to healthy controls (35). Pinocembrin exhibits potent antioxidant effects, which directly remove ROS *via* its phenolic structures and enhance antioxidant defense *via* its phenolic and non-phenolic structures (36).

Oxidative stress primarily promotes arrhythmia through electrical remodeling and structural remodeling. ROS leads to Ca^2+^ overload by activating CaMKII, which is a recognized sensor of Ca^2+^ and ROS (37). H2O2 is a ROS that causes cardiomyocyte damage and electrical remodeling, including prolonged cardiac action potential, increased late I_Na_, early after depolarizations (EADs), and sodium and calcium overload, which contribute to the development of cardiac pathology (38). Previous research revealed that oxidative stress suppressed the content of Cx40 and Cx43 in the LA and resulted in abnormalities in the coupling of gap junctions, which were recovered by antioxidants (39). ROS leads to apoptosis by activating the stress-inducible p38MAPK pathway, and treatment with SB203580 (an inhibitor of p38MAPK) or antioxidants reduce apoptosis (40). Reduced levels of H2O2 promote p38MAPK activation and protein synthesis, and high levels of H2O2 stimulate p38MAPK to induce apoptosis (41). ROS contributes to the development of fibrosis by promoting the content of TGF-β1, and TGF-β1 induces the production of ROS, which provides a basis for arrhythmia (42).

NADPH oxidase (NOX) contributes to the generation of ROS in patients with AF, and NOX2 and NOX4 are primarily present in the heart (43). NOX2 and NOX4 are present in endothelial cells, cardiomyocytes, and fibroblasts, and NOX4 is present in vascular smooth muscle cells. Overexpression of NOX generates an arrhythmogenic phenotype mediated by ROS. NOX4 increases the expression of ROS-induced TGF-β1 to promote cardiac myofibroblast differentiation and promotes cardiomyocyte apoptosis *via* the NOX4/ROS/p-p38 pathway (44, 45). Angiotensin II (AngII) promotes myocardial hypertrophy and fibrosis *via* NOX2, and continuous AngII stimulation triggers cardiomyocyte apoptosis *via* the NOX2/ROS/CaMKII pro-apoptotic pathway (46). Glutathione (GSH) regulates antioxidant defense by clearing free radicals and other reactive oxygen species and is oxidized to GSSG during the process (47). GSH/GSSG reflects the antioxidative ability of the body. SOD protects cells from external stimulation at the earliest phases and removes MDA, which is an indicator of the degree of lipid peroxidation in cell membranes (48).

Our study showed that oxidative stress was promoted in depressed rats and was ameliorated by pinocembrin administration. Pinocembrin also inhibited the ROS/p-p38MAPK pro-apoptotic pathway and the ROS/TGF-β1 pro-fibrotic pathway in a rodent model of depression.

## Conclusion

The current research primarily demonstrated that pinocembrin ameliorated autonomic imbalance, atrial electrical disorder, a lack of ion channels and gap junctions, atrial cell apoptosis, atrial fibrosis, and oxidative stress, which reduced AF susceptibility in the depressed rats. Pinocembrin inhibited the ROS/p-p38 pathway to reduce depression-induced apoptosis and inhibited the ROS/TGF-β1 pathway to reduce depression-induced fibrosis. These findings demonstrate that pinocembrin is a therapeutic strategy with great promise for the treatment of AF in patients with depression.

## Limitations

Our study demonstrated that pinocembrin decreased AF susceptibility in a rodent model of depression. However, there are still several limitations. CUMS is not the only method to construct depression models. Therefore, depression models constructed using other methods should also be used to confirm our results. Pinocembrin ameliorated oxidative stress in depressed rats, but other mechanisms (such as inflammation) also play a role in depression-induced AF, which requires further investigation.

## Data Availability Statement

The original contributions presented in the study are included in the article, further inquiries can be directed to the corresponding author/s.

## Ethics Statement

Animal care and experiments were performed based on the Guide for Care and Use of Laboratory Animals and were approved by the Animal Ethics Committee of Wuhan Third Hospital, China (Animal Ethical Number: SY2020-027-1).

## Author Contributions

QR, CZ, and TY designed this study. QR, XC, TY, YS, and XZ performed the experiments. QR, XC, and WW collected and analyzed the data. QR drafted the first manuscript. CZ and SS revised the manuscript. BY and QZ determined the final manuscript. All the authors contributed to the study and approved the final manuscript.

## Conflict of Interest

The authors declare that the research was conducted in the absence of any commercial or financial relationships that could be construed as a potential conflict of interest.

## Publisher’s Note

All claims expressed in this article are solely those of the authors and do not necessarily represent those of their affiliated organizations, or those of the publisher, the editors and the reviewers. Any product that may be evaluated in this article, or claim that may be made by its manufacturer, is not guaranteed or endorsed by the publisher.
